# A Machine Learning-Based Hypoxia-Related Gene Signatures to Facilitate Prediction of Cetuximab Response in Patients with Colorectal Cancer

**DOI:** 10.7150/ijms.114833

**Published:** 2025-08-11

**Authors:** Cuizhen Zhang, Wanjie Niu, Jiangtao Zhang, Yingyi Zheng, Zhiru Chen, Fali Zhang, Xiaoyan Qiu

**Affiliations:** 1 Department of Pharmacy, Huashan Hospital, Fudan University, 12 Middle Urumqi Road, Shanghai, 200040, China.; 2 Key Laboratory of Big Data Intelligent Computing, Chongqing University of Posts and Telecommunications, Chongqing, 400065, China.; 3 Department of Pharmacy, The Affiliated Guangdong Second Provincial General Hospital of Jinan University, 466 Middle Xingang Road, Guangzhou, 510317, China.

**Keywords:** colorectal cancer, hypoxia-related genes, cetuximab, K-nearest neighbors, machine learning models

## Abstract

**Background** There is significant individual variation in the efficacy of cetuximab for the treatment of colorectal cancer (CRC). However, effective models to predict treatment outcomes are still lacking in clinical practice.

**Methods** Datasets (GSE106582 and GSE83889) were used to identify differentially expressed genes (DEGs) in CRC by the 'Limma' package in R software. Hypoxia-related genes were retrieved from the Molecular Signatures Database and cross-referenced with CRC DEGs. Protein expression levels were verified using immunohistochemistry (IHC) data from the Human Protein Atlas (HPA), and prognostic significance was assessed through the Kaplan-Meier plotter platform. Additionally, pathway and immune infiltration analyses were performed using the GSCA platform. We also successfully constructed a prediction model for cetuximab treatment response using the K-nearest neighbors (KNN) algorithm in GSE108277 dataset, in which the feature selection was performed through the permutation importance method.

**Results** Analysis of GSE106582 and GSE83889 identified 417 overlapping DEGs by comparing cancer tissues with normal controls, including 16 hypoxia-related genes. 6 genes (*BGN*, *DDIT4*, *MIF*, *SLC2A1*, *STC2*, and *TGFBI*) were upregulated, and 10 genes (*CA12*, *CITED2*, *MT1E*, *MT2A*, *NEDD4L*, *PCK1*, *PLAC8*, *PPARGC1A*, *SELENBP1*, and *SRPX*) were downregulated in CRC. Survival analysis revealed that the 16 hypoxia-related DEGs were linked to the survival outcomes of CRC patients. Pathway analysis indicated that these genes were almost involved in EMT, cell cycle, and RTK pathways. Furthermore, these genes play a role in the infiltration of immune cells and may regulate the immune microenvironment. A prediction model for cetuximab response was developed, based on 10 key genes (*CA12*, *DDIT4*,* MIF*, *MT2A*, *NEDD4L*, *PLAC8*, *SELENBP1*, *SLC2A1*, *SRPX*, and *TGFBI*) and dataset from GSE108277. The model demonstrated robust performance with an accuracy of 0.9500, precision of 0.8378, recall of 1.0000, F1-score of 0.9118, and a receiver operating characteristic-area under the curve (ROC-AUC) of 0.9663.

**Conclusion** Our study identifies 10 hypoxia-related DEGs as key players in CRC progression and cetuximab response. And we successfully developed a predictive model to forecast the response of CRC patients to cetuximab treatment. This study will provide valuable biomarkers for CRC prognosis and help guide more effective therapeutic strategies.

## Introduction

Colorectal cancer (CRC) is the third most common cancer and the fourth leading cause of deaths related to cancer worldwide. In China, both the incidence and mortality rates of CRC are on the rise, currently ranking as the second and fourth most common malignancy, respectively 1, 2. Despite advancements in diagnostic techniques and therapeutic strategies, significant inter-patient variability in disease progression contributes to the poor prognosis of CRC patients 2. Therefore, the selection of individualized treatment regimens and the evaluation of prognosis for CRC patients remains a challenge.

The current treatment of CRC primarily involves a combination of surgery, radiotherapy, chemotherapy, and targeted therapy, surgical resection remains the most effective curative approach for localized CRC patients. In contrast, patients with advanced or metastatic CRC rely heavily on systemic treatments 3. In recent years, tyrosine kinase inhibitors (TKIs) targeted therapies have brought new insights into therapeutic options for patients 4. Among these, the epidermal growth factor receptor (EGFR) -target therapy remains central pillar in the molecular targeted treatment of CRC. EGFR plays a pivotal role in the initiation, progression, and metastasis of tumor cells by activating many downstream pathways in CRC, such as RAS/RAF/MEK/ERK and PI3K/AKT/mTOR pathway 5. EGFR-target therapy (cetuximab and panitumumab) and small-molecule TKIs have been reported to inhibit tumor growth and disease progression, which demonstrate the promising therapeutic option in CRC patients 6.

Cetuximab is a monoclonal antibody targeting EGFR to block tumor cell growth and proliferation. It is widely used in the treatment of advanced and metastatic CRC. Treatment with cetuximab is recommended for patients with KRAS wild-type metastatic CRC, particularly for those with left-sided primary tumors. Furthermore, cetuximab is frequently administered in conjunction with chemotherapy protocols to bolster therapeutic effectiveness. Researches have shown that the combination of cetuximab with chemotherapy markedly enhances progression-free survival (PFS) and overall survival (OS) among CRC patients 7, 8. However, the efficacy of cetuximab is substantially limited by the emergence of resistance over time 9. Additionally, individual differences in drug response remain a key factor influencing the prognosis of CRC patients. Studies have shown that EGFR mutations, BRAF mutations, and changes in the tumor microenvironment were closely associated with cetuximab resistance 10. However, the main mechanisms underlying cetuximab resistance remain unclear.

Hypoxia is a common feature in tumor and is recognized as an important factor contributing to cancer development and drug resistance 11. Hypoxia-inducible factor 1-alpha (HIF-1α) typically increased and activated epithelial-to-mesenchymal transition (EMT) under hypoxic conditions, which regulated the proliferation and activation of cancer stem cells (CSCs) 12. Cetuximab effectively reverses the Warburg effect by inhibiting the HIF-1-mediated glycolytic process in cancer cells, thereby significantly suppressing their metabolic activity 13. Previous studies have shown that hypoxia-related genes mediate cetuximab treatment response through various molecular mechanisms 14, 15. In the treatment of head and neck squamous cell carcinomas (HNSCC), cetuximab demonstrates increased sensitivity under hypoxic conditions. Additionally, cetuximab treatment can partially reverse hypoxia-induced EMT and the expression of stem cell markers in HNSCC cells 14. In CRC research, it has been demonstrated that circHIF1A promotes HIF1α-mediated metabolic changes, thereby inducing cetuximab resistance in CRC cells 16. Furthermore, hypoxia-driven metabolic reprogramming, including increased glycolysis and lactate production, enhances tumor cell survival under stress conditions 17. These metabolic adaptations coupled with activation of PI3K/AKT and RTK pathways can synergize with EGFR signaling to further weaken cetuximab's antitumor effects 18, 19. Therefore, thorough research into the aberrant expression of hypoxia-related genes and their mechanisms in CRC is crucial for enhancing the therapeutic efficacy of EGFR inhibitors such as cetuximab.

The application of machine learning in personalized treatment is becoming increasingly widespread, with commonly used algorithms including k-nearest neighbors (KNN), support vector machines (SVM), Random Forests (RF), Neural Networks, and Gradient Boosting Trees. Machine learning has shown significant value in treatment decision-making and prognostic prediction for CRC. The COLOXIS AI model, developed by the University of Pittsburgh, precisely identifies patients who would benefit from oxaliplatin-based adjuvant chemotherapy by analyzing gene expression profiles of tumor tissues 20. The model has helped avoid unnecessary side effects in approximately half of the patients and significantly improved the benefit-risk ratio of treatment, providing important decision support for clinical practice. The MUSK model from Stanford University combines medical imaging and textual data to accurately predict survival rates and immune therapy responses for 16 types of cancer, including CRC 21. Meanwhile, a research team from Zhejiang University identified macrophage-centered immune modules to predict chemotherapy responses and guide personalized treatment using multi-omics analysis and deep learning models 22. Therefore, the application of machine learning in the treatment of CRC is of great significance for achieving personalized treatment, improving patients' quality of life, and enhancing treatment outcomes.

This study aims to develop a machine learning-based model capable of predicting the cetuximab response of CRC patients. We intend to identify the key biomarkers that influence cetuximab efficacy by integrating hypoxia-related gene expression data with the genomic information of CRC patients. We also evaluated the predictive accuracy and interpretability of the model in clinical practice. The proposed model was expected to enhance therapeutic outcomes and provide critical support for clinical decision-making, thereby advancing the personalization of CRC treatment.

## Methods

### Data sources

To investigate the genomic features associated with CRC and cetuximab efficacy, three publicly available datasets were retrieved from the Gene Expression Omnibus (GEO, http://www.ncbi.nlm.nih.gov/geo/) database. The datasets GSE106582, GSE83889, and GSE108277 were generated using the GPL10558 microarray platform, which provides a unified methodological foundation to maintain consistency throughout the data preprocessing and analysis stages. The datasets are detailed as follows: (I) GSE106582: This dataset encompasses a total of 194 samples. For the purposes of this study, 68 CRC tissue samples and their corresponding 68 normal mucosal control samples were chosen for differential expression analysis. (II) GSE83889: This dataset consists of 136 samples, which include 35 normal tissue samples and 101 CRC tissue samples. We identified differentially expressed genes (DEGs) in CRC tissues compared to normal tissues with the GSE106582 and GSE83889 datasets. (III) GSE108277: This dataset comprises 120 samples with comprehensive cetuximab efficacy data. It was utilized to train and validate machine learning models designed to forecast cetuximab treatment responses, thereby aiding in the identification of biomarkers associated with drug sensitivity and resistance.

Hypoxia-related genes were extracted from the hallmark gene set in the Molecular Signatures Database v7.0 (MSigDB, https://www.gsea-msigdb.org/gsea/msigdb). This hallmark gene set consists of 200 genes that are directly or indirectly involved in hypoxia regulation pathways, identified from the literature available in PubMed and other biological public databases 23.

All datasets used in this study are publicly accessible and free to use. The study adhered strictly to data access policies and publication guidelines. Consequently, ethical approval from an Ethics Committee was not required.

### Identify the differential genes significantly associated with CRC

The datasets GSE106582 and GSE83889 were obtained from the GEO database. Differential expression analysis of mRNAs was conducted using the “Limma” package (version 3.40.2) in R software (version 4.0.3). To minimize false positives, we applied Benjamini and Hochberg (BH) adjustment for statistical correction of p, controlling the false discovery rate (FDR). DEGs were identified based on the criteria: adjusted p < 0.05 and |logFC| > 1.

To further explore the potential functions of the identified DEGs, functional enrichment analyses were performed. Gene ontology (GO) annotation was utilized to examine gene functions across three domains: Molecular Function (MF), Biological Process (BP), and Cellular Component (CC). Additionally, Kyoto encyclopedia of genes and genomes (KEGG) pathway enrichment analysis was conducted to investigate high-level functional insights and genomic pathways associated with the target genes. Both GO and KEGG analyses were carried out using the “ClusterProfiler” package in R software, with a significance threshold set at p < 0.05.

### Acquisition of Hypoxia-Related DEGs associated with CRC

To investigate the involvement of hypoxia-related genes in CRC, the results from the differential expression analyses of GSE106582 and GSE83889 were compared to identify overlapping genes. These overlapping DEGs were further matched with a curated list of 200 hypoxia-related genes from MSigDB v7.0, involving genes in hypoxia regulation pathways. This cross-referencing process identified a subset of hypoxia-related genes that were both dysregulated in CRC and linked to hypoxic processes, offering key candidates for further analysis and validation.

### Verification of protein expression levels of Hypoxia-Related DEGs in CRC

The Human Protein Atlas (HPA, https://www.proteinatlas.org/) database was employed to obtain immunohistochemistry (IHC) results for the proteins of interest 24. The HPA database provides high-quality IHC images, allowing the visualization and confirmation of protein expression patterns in CRC tissues compared to normal tissues.

### Differential analysis of hypoxia-related DEGs in different pathological stages of CRC

GEPIA (http://gepia2.cancer-pku.cn/#index) platform was utilized to analyze the expression of hypoxia-related genes and their correlation with prognosis across different pathological stages in CRC patients 25. We applied log2 transformation to the RNA-seq data to ensure that the transcript per million values followed a normal distribution assumption. Subsequently, we performed one-way analysis of variance to compare gene expression levels across different pathological stages, with a significance threshold set at p < 0.05.

### The Association between Hypoxia-Related DEGs and the prognosis of CRC Patients

The prognostic significance of hypoxia-related DEGs in CRC was evaluated using Kaplan-Meier plotter (https://kmplot.com/analysis/) to assess their association with OS, relapse-free survival (RFS), and post-progression survival (PPS) 26, 27. The analysis for OS was conducted using data from 1,061 CRC patients, for RFS with 1,336 CRC patients, and for PPS with 311 CRC patients. The CRC patients were stratified into high and low expression groups based on the best percentile cutoff automatically selected by the Kaplan-Meier plotter platform. For each survival analysis, the Affymetrix IDs for each gene were used. The log-rank test was used to evaluate the impact of gene expression on prognosis by calculating the hazard ratio (HR) and logrank p, with p < 0.05 considered statistically significant.

### The correlation between Hypoxia-Related DEGs with pathways and immune infiltration in CRC

We employed two complementary approaches to evaluate the functional roles of the 16 hypoxia-related DEGs in CRC. For pathway analysis, the effects of individual gene expression levels on pathway activity were assessed by calculating pathway activity scores (PAS) for ten key cancer-related pathways using reverse phase protein array data from the TCGA database. Differences in PAS between high- and low-expression groups for each gene were evaluated using student's t-test, p-values adjusted by FDR. Genes were classified as pathway activators or inhibitors based on whether their high expression increased or decreased pathway activity, respectively. For immune infiltration analysis, the infiltration levels of 24 immune cell types were estimated using ImmuCellAI, based on predefined gene set signatures. The mRNA expression levels of individual genes were correlated with immune cell infiltration levels using Spearman correlation analysis, with p-values adjusted by FDR. The above analyses were conducted on the GSVA (https://guolab.wchscu.cn/GSCA/#/) platform to evaluate the functional roles of the 16 hypoxia-related DEGs in CRC 28, 29.

### Screening of Hypoxia-Related DEGs associated with cetuximab response and construction of a treatment response prediction model

We employed several classical machine learning algorithms for predictive modeling, assessing their performance using a 10-fold cross-validation framework. Each model was evaluated without hyperparameter tuning to ensure a fair and unbiased comparison of their stability and feasibility. The dataset, consisting of 120 samples and 16 features, was randomly partitioned into 70% training and 30% validation subsets for each iteration. Stratified sampling was applied to preserve class distribution within both the training and validation sets. Cross-validation was performed such that data splitting was independently randomized for each fold to enhance robustness and minimize overfitting. Hyperparameter tuning was conducted exclusively within the training subsets to avoid data leakage and preserve the integrity of the validation process. Model performance was assessed using various metrics, including accuracy, F1-score, precision, recall, and receiver operating characteristic-area under the curve (ROC-AUC) score.

## Results

### Identification of significant DEGs and functional enrichment analysis in CRC

**Figure 1:**
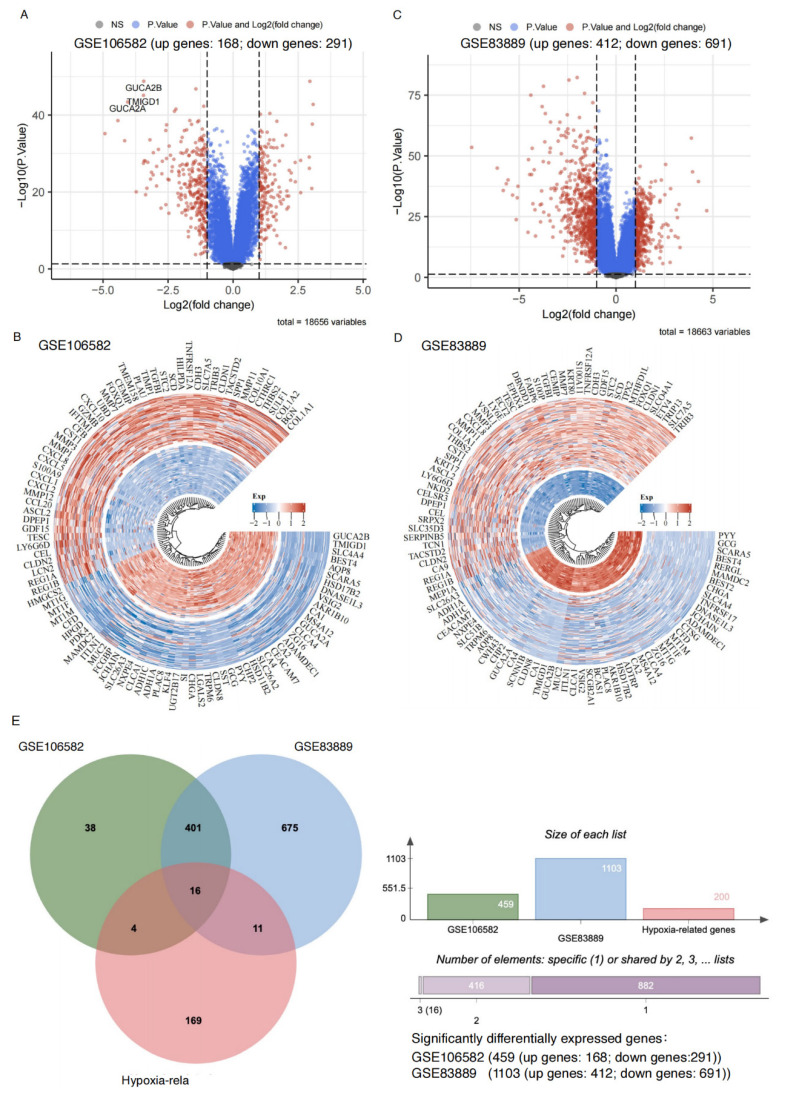
** Sixteen hypoxia-related genes significantly differentially expressed in CRC. A**: Volcano plot illustrating gene expression differences in the GSE106582 dataset. The x-axis represents log2 fold change (FC) in gene expression, while the y-axis shows statistical significance (-log10 p-value). Gray dots: Genes that do not meet the significance threshold (non-significant, high P-value). Blue dots: Genes with significant p-values but without substantial fold changes (|logFC| ≤ 1). Red dots: Genes that are both statistically significant (adjusted p-value < 0.05) and exhibit notable expression changes (|logFC| > 1), indicating significant upregulation or downregulation. **B**: Circular heatmap of differentially expressed genes in the GSE106582 dataset. Red: Upregulated genes in colorectal cancer. Blue: Downregulated genes in colorectal cancer. **C**: Volcano plot for the GSE83889 dataset, following the same criteria as in panel A. **D**: Circular heatmap displaying differentially expressed genes in the GSE83889 dataset, similar to panel B. **E**: Venn diagram comparing differentially expressed genes between the GSE83889 and GSE106582 datasets, highlighting the overlap with hypoxia-related genes.

We analyzed datasets GSE106582 and GSE83889, a total of 459 and 1,103 significant DEGs were identified, respectively (adjusted p < 0.05, |logFC| > 1). Among these, 168 upregulated and 291 downregulated genes were identified in GSE106582 (Figure 1A-B, Supplemental Table 1), while GSE83889 included 412 upregulated and 691 downregulated genes (Figure 1C-D, Supplemental Table 2).

Functional enrichment analysis revealed distinct characteristics of upregulated and downregulated genes in both datasets. In GSE106582 (Supplemental Table 3), upregulated genes were associated with extracellular matrix organization, cell chemotaxis, and collagen metabolism (Supplemental Figure 1A), as well as enriched molecular functions such as chemokine receptor binding and cytokine activity. KEGG pathway analysis highlighted PI3K/AKT signaling, ECM-receptor interaction, and NF-kB signaling (Supplemental Figure 1B). Downregulated genes were linked to biological processes such as metal ion response, hormone metabolism, and stress response, with suppressed oxidoreductase activity and altered membrane transport (Supplemental Figure 1C-D). Key metabolic pathways, including fatty acid degradation and retinol metabolism, were also enriched.

Similarly, in GSE83889 (Supplemental Table 4), upregulated genes were primarily involved in cell cycle-related processes such as ribosome biogenesis, mitotic nuclear division, and chromosome segregation, contributing to structural components like the mitotic spindle and extracellular matrix (Supplemental Figure 2A). Downregulated genes were enriched in lipid metabolism, immune functions such as MHC protein complex, and processes related to cytokine interactions (Supplemental Figure 2B). KEGG analysis further revealed upregulated genes were enriched in tumor-related pathways, including PI3K/AKT signaling, cell cycle, and DNA replication, while downregulated genes were associated with suppressed immune pathways and reduced metabolic activity (Supplemental Figure 2C, D).

### Verification of Hypoxia-Related DEGs in CRC and Protein Expression Levels

A total of 417 overlapping DEGs were identified between GSE106582 (459 DEGs) and GSE83889 (1,103 DEGs) (Figure 1E). These 417 DEGs were further intersected with a predefined list of 200 hypoxia-related genes, resulting in the identification of 16 hypoxia-related DEGs (Figure 1E). Among these, 6 genes (BGN, DDIT4, MIF, SLC2A1, STC2, and TGFBI) were upregulated, while 10 genes (CA12, CITED2, MT1E, MT2A, NEDD4L, PCK1, PLAC8, PPARGC1A, SELENBP1, and SRPX) were downregulated at the mRNA expression level.

**Figure 2:**
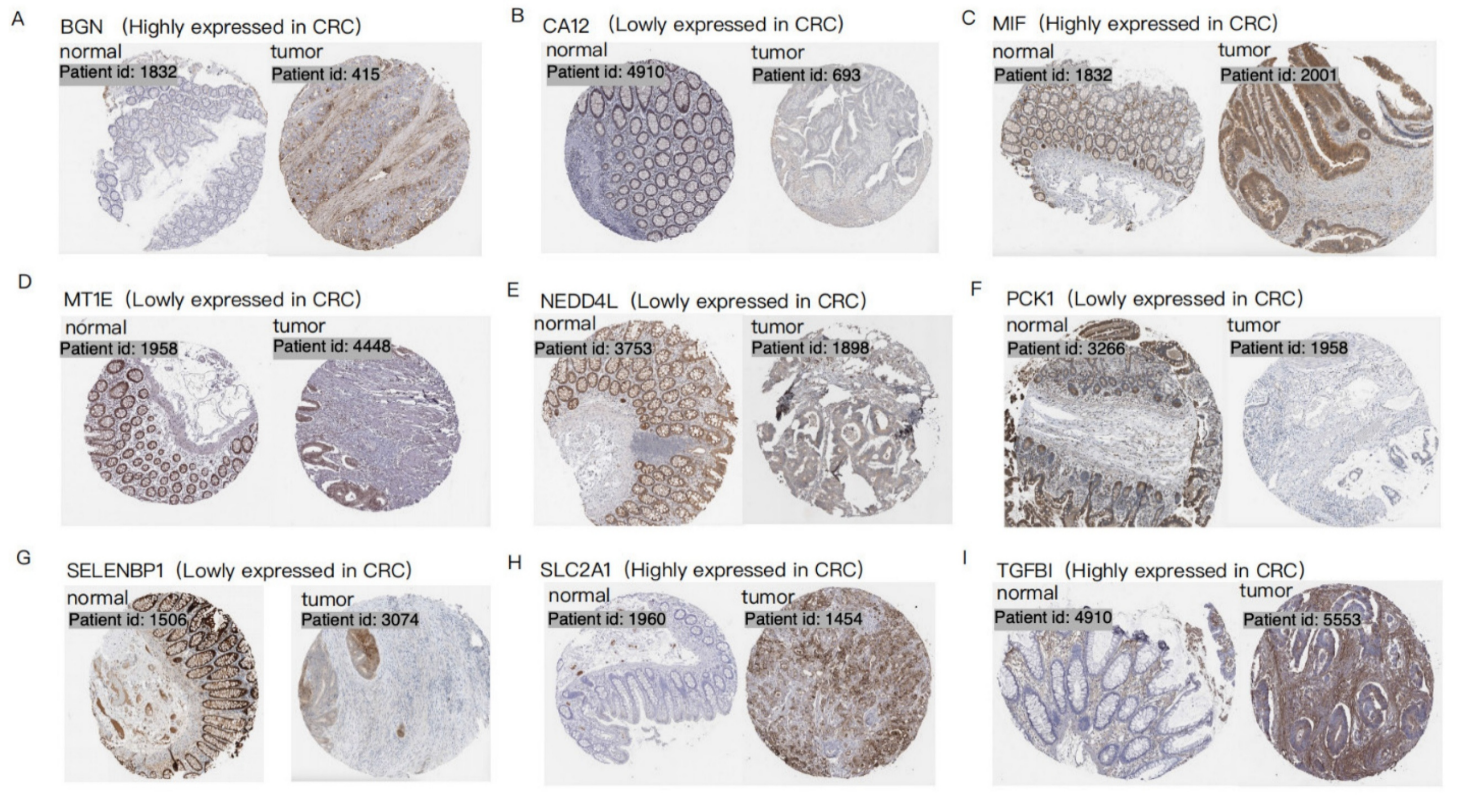
** Immunohistochemistry of 9 hypoxia-related DEGs in CRC.** Each set of images shows the difference in expression of a specific gene in normal tissue and CRC tumor tissue, visualized by tissue section staining technology. Brown represents the expression location of the target gene. Each panel (**A-I**) represents a distinct gene: (**A**)BGN; (**B**) CA12; (**C**) MIF; (**D**) MT1E; (**E**) NEDD4L; (**F**) PCK1; (**G**) SELENBP1; (**H**) SLC2A1; (**I**) TGFBI.

Protein level analysis further validated these findings, demonstrating strong protein expression for upregulated genes and weak or negligible expression for downregulated genes. IHC data from the HPA database provided robust support for the differential expression patterns of 9 hypoxia-related DEGs (BGN, CA12, MIF, MT1E, NEDD4L, PCK1, SELENBP1, SLC2A1, and TGFBI) in CRC patients, consistent with observations at both mRNA and protein levels (Figure 2). Data for the remaining 7 genes (CITED2, DDIT4, MT2A, PLAC8, PPARGC1A, SRPX, and STC2) were unavailable in the HPA database.

### Hypoxia-Related DEGs correlated with the pathological stage of CRC

**Figure 3:**
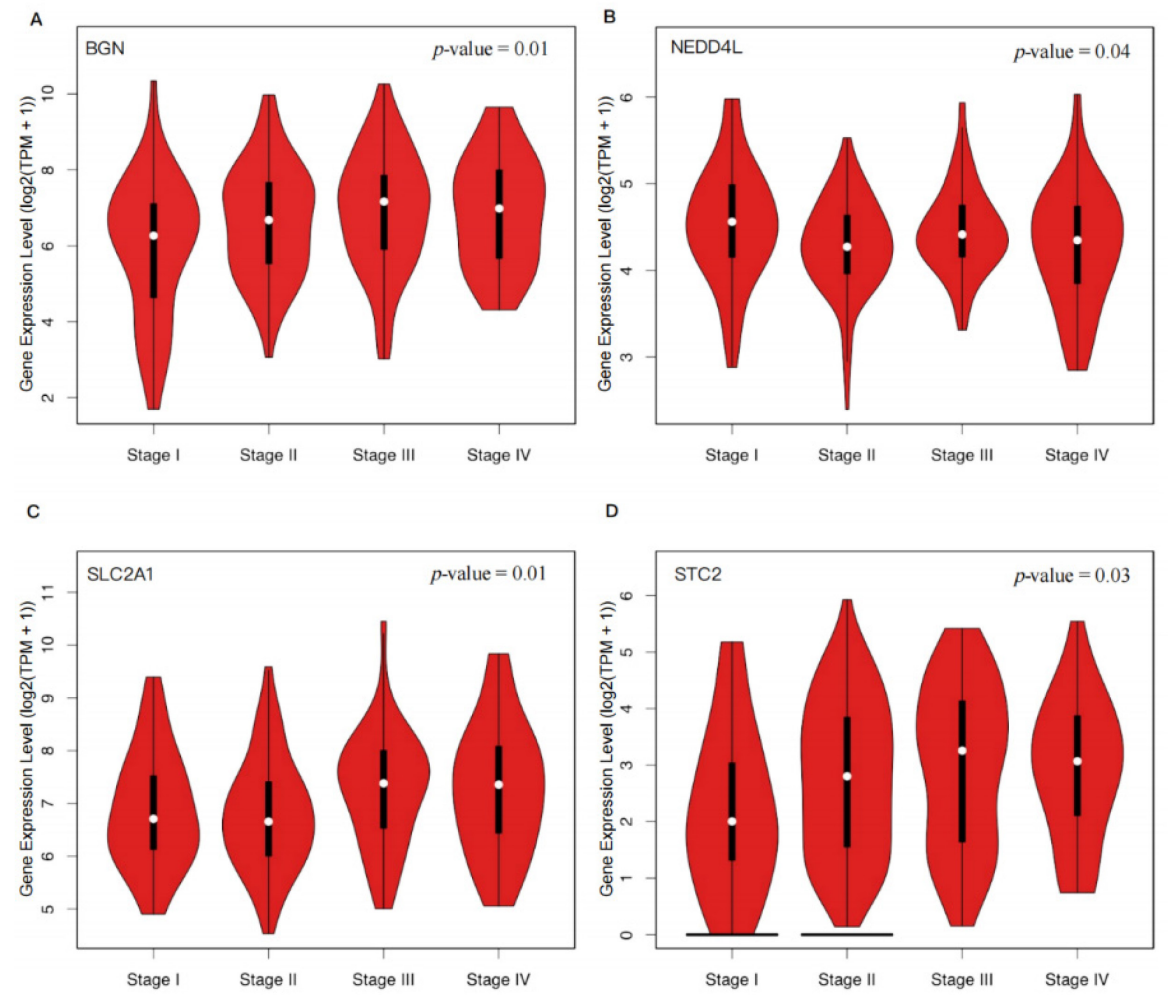
**Expression distribution of four hypoxia-related DEGs in different pathological stages of CRC.** The violin plots show the expression distribution of four hypoxia-related DEGs in 275 CRC tissue samples across different pathological stages. The y-axis represents the log2(TPM+1) transformed gene expression, and the x-axis corresponds to the clinical stages (Stage I, Stage II, Stage III, Stage IV) of CRC. Each plot's thick black line represents the median gene expression, while the white dots indicate the distribution density of the gene expression data. A p-value < 0.05 was used to assess the statistical significance of the difference in gene expression between the stages. (**A**) BGN; (**B**) NEDD4L; (**C**) SLC2A1; (**D**) STC2.

Through our analysis, we found that the expression levels of BGN (p = 0.01), SLC2A1 (p = 0.01), and STC2 (p = 0.03) significantly increased with the progression of CRC, while the expression level of NEDD4L (p = 0.04) decreased (Figure 3). These findings suggested that upregulation of BGN, SLC2A1, and STC2 and downregulation of NEDD4L may promote the proliferation, invasion, and metastasis of tumor cells in CRC.

### Hypoxia-Related DEGs correlated with prognostic significance in CRC

**Figure 4:**
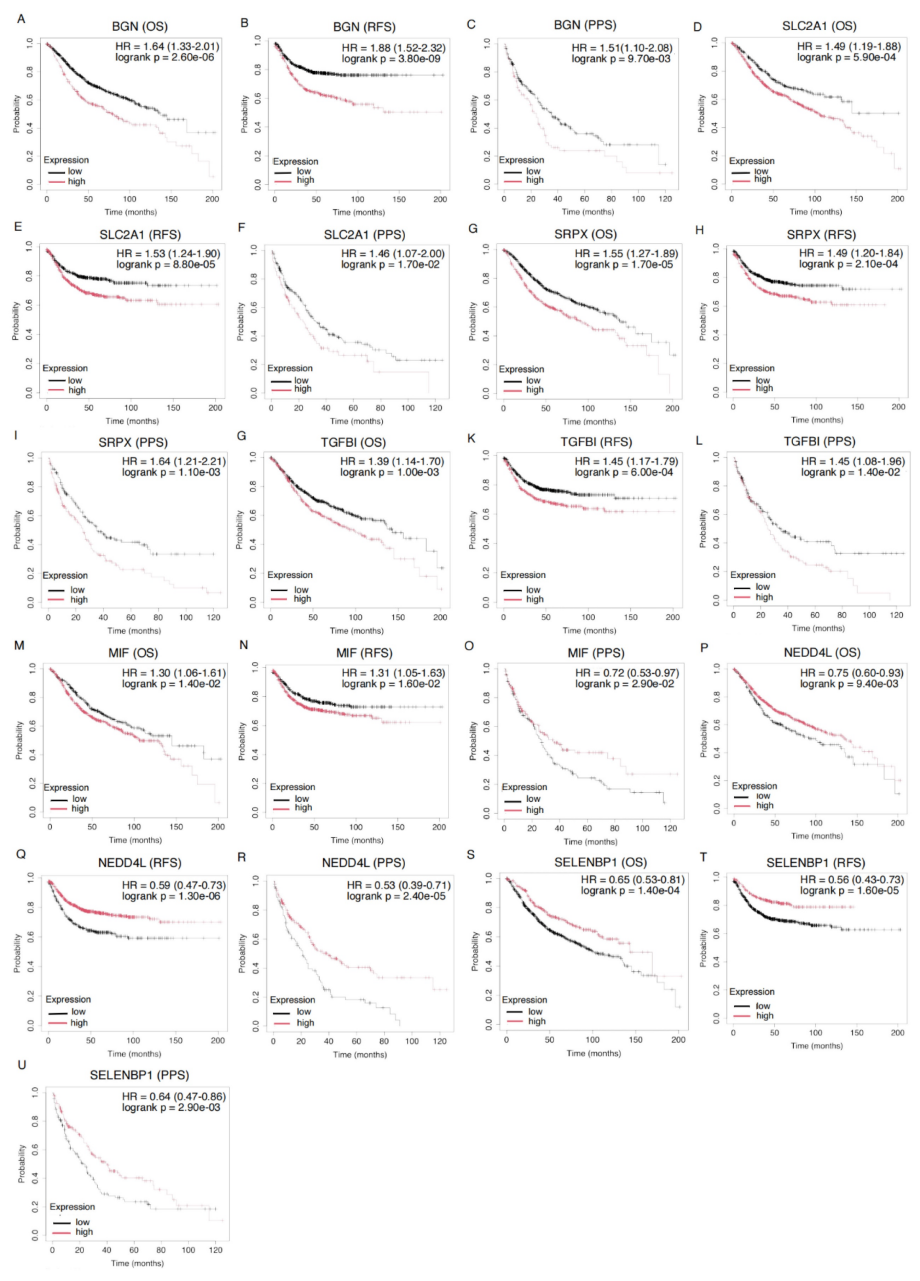
** Kaplan-Meier Survival Curves for Gene Expression and Their Prognostic Impact on OS, RFS, and PPS in CRC Patients.** Kaplan-Meier survival analysis of the genes BGN (201261_x_at), SLC2A1 (201249_at), SRPX (204955_at), TGFBI (201506_at), MIF (221262_s_at), NEDD4L (212445_s_at), and SELENBP1 (214433_s_at) across different survival outcomes: OS, RFS, and PPS. Each plot compares survival probabilities between high and low expression groups. HR and corresponding p-values are displayed for each gene, with statistical significance indicated (logrank p < 0.05). The black lines represent the low expression group, while the red lines represent the high expression group.

We analyzed the prognostic significance of 16 hypoxia-related DEGs in CRC. All genes significantly influenced the prognosis of CRC patients (p < 0.05). Seven genes (BGN, MIF, NEDD4L, SELENBP1, SLC2A1, SRPX, and TGFBI) were associated with prognosis in OS, RFS, and PPS (Figure 4, Supplemental Table 5).

High expression of BGN correlated with poor prognosis, with HRs of 1.64 for OS, 1.88 for RFS, and 1.51 for PPS. Similarly, high expression of SLC2A1 (OS: HR = 1.49, RFS: HR = 1.53, PPS: HR = 1.46) and SRPX (OS: HR = 1.55, RFS: HR = 1.49, PPS: HR = 1.64) was associated with worse prognosis. Elevated TGFBI expression was linked to poor prognosis in both OS, RFS, and PPS (OS: HR = 1.39, RFS: HR = 1.45, PPS: HR = 1.45), further supporting its role in CRC prognostication. MIF exhibited a mixed pattern: high expression was linked to poor prognosis in OS and RFS (OS: HR = 1.30, RFS: HR = 1.31), while low expression was associated with worse prognosis in PPS (PPS: HR = 0.72), indicating distinct biological functions at different CRC stages. In contrast, low expression of NEDD4L (OS: HR = 0.75, RFS: HR = 0.59, PPS: HR = 0.53) and SELENBP1 (OS: HR = 0.65, RFS: HR = 0.56, PPS: HR = 0.64) was associated with poor prognosis, suggesting a protective role by inhibiting tumor progression.

Further analysis identified other genes significantly impacting CRC prognosis. CITED2, DDIT4, MT2A, PLAC8, and STC2 were significantly associated with OS (Supplemental Figure 3, Supplemental Table 5). CITED2 (HR = 1.24) and DDIT4 (HR = 1.43) were linked to poor OS, while MT2A (HR = 1.25) and STC2 (HR = 1.25) were associated with worse OS. PLAC8 was linked to better OS (HR = 0.77). Moreover, CITED2, DDIT4, MT1E, PPARGC1A, and STC2 were significantly associated with RFS, suggesting their roles in CRC recurrence and metastasis. Additionally, CA12, PCK1, and PPARGC1A were significantly associated with PPS, highlighting their prognostic value in advanced-stage patients (Supplemental Figure 3, Supplemental Table 5).

### Hypoxia-Related DEGs regulated the diverse pathways in CRC

**Figure 5:**
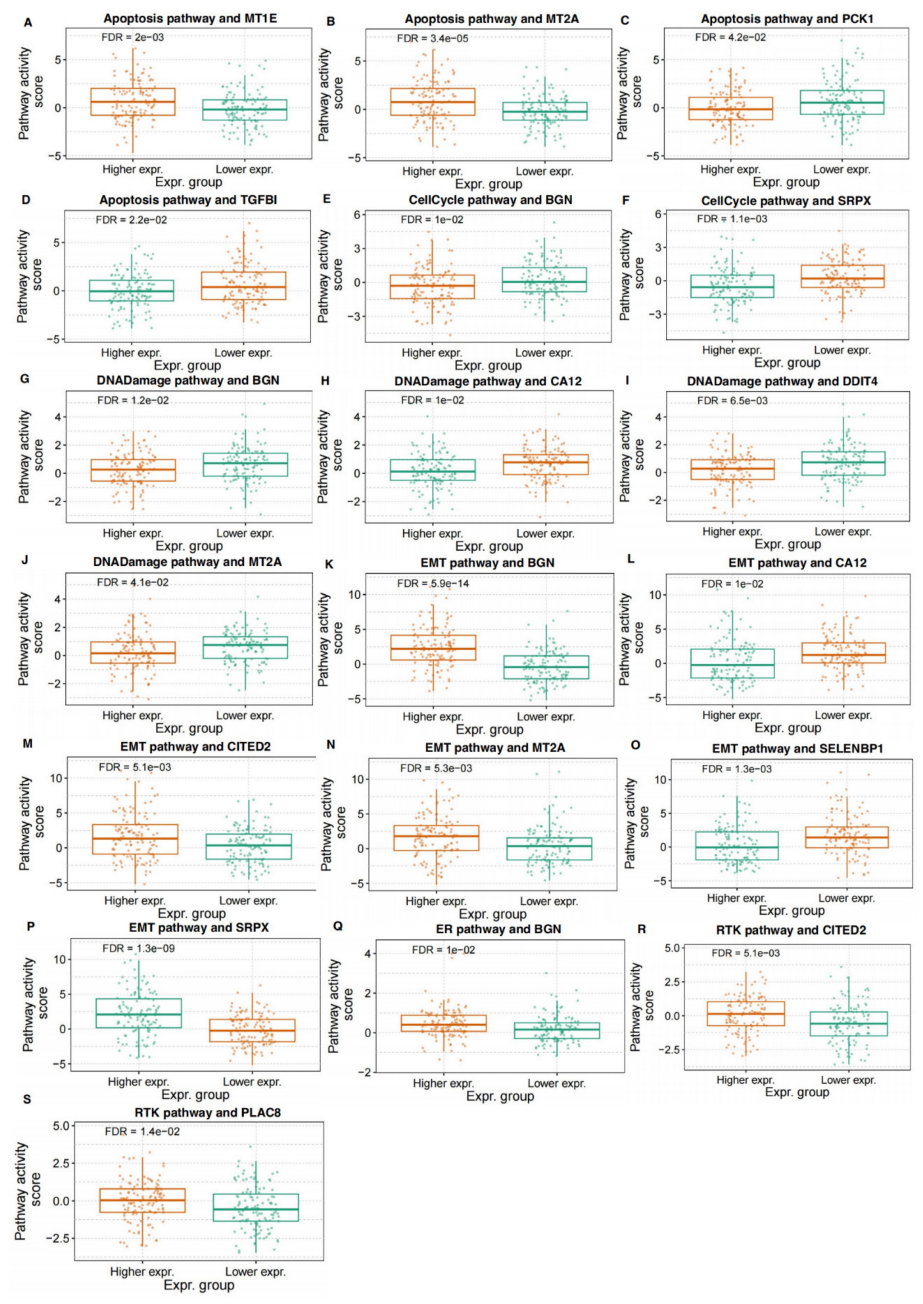
** Association of Hypoxia-Related DEGs with Pathway Activity in CRC.** The figure illustrates the correlation between the expression of hypoxia-related DEGs and the activity of key biological pathways in CRC. Panels (**A-D**) show the relationship between the expression of MT1E, MT2A, PCK1, and TGFB1 with the activity of the apoptosis pathway. Panels (**E**,** F**) display the association of BGN and SRPX expression with the cell cycle pathway activity. In panels (**G-J**), the expression of BGN, CA12, DDIT4, and MT2A is correlated with the activity of the DNA damage pathway. Panels (**K-P**) present the correlation of BGN, CITED2, MT2A, SRPX, CA12, and SELENBP1 with the EMT pathway activity. Panels (**Q**,** R**) show the relationship between CITED2 and PLAC8 expression and the RTK signaling pathway activity. Panel (**S**) depicts the correlation of BGN expression with hormone-related pathway activity.

We analyzed the association of hypoxia-related DEGs' expressions with the activity of various biological pathways in CRC. The results indicated that the expression levels of multiple genes were significantly correlated with pathway activity scores in pathways related to the cell cycle, DNA damage, EMT, RTK signaling, and hormone-related pathways. Specifically, in the apoptosis pathway, higher expression of MT1E and MT2A was significantly associated with increased pathway activity, while higher expression of PCK1 and TGFB1 was associated with decreased activity (Figure 5A-D). In the cell cycle pathway, higher expression of both BGN and SRPX was correlated with decreased pathway activity, suggesting their potential regulatory roles in cell cycle control (Figure 5E, F). Furthermore, higher expression of BGN was associated with decreased DNA damage pathway activity, while higher expression of CA12, DDIT4, and MT2A was correlated with decreased activity (Figure 5G-J). In the EMT pathway, higher expression of BGN, CITED2, MT2A, and SRPX was correlated with increased pathway activity, whereas higher expression of CA12 and SELENBP1 was associated with decreased activity (Figure 5K-P). In the RTK signaling pathway, higher expression of CITED2 and PLAC8 was associated with increased pathway activity (Figure 5Q, R). Additionally, higher expression of BGN was associated with increased activity in hormone-related pathways (Figure 5S). These correlative findings suggest the potential involvement of these specific hypoxia-related DEGs in modulating key biological pathways in CRC.

### Hypoxia-related DEGs were associated with immune cell infiltration in CRC

In the immunological correlation analysis of hypoxia-related DEGs, different genes exhibited specific associations with various immune cell types in CRC, with all correlations showing at least moderate strength (correlation coefficients greater than 0.3) (Supplemental Figure 4, Supplemental Table 6). Regarding B cells, BGN, DDIT4, MIF, TGFBI, and STC2 showed negative correlations, suggesting that these genes might have suppressed B cell infiltration or function in a hypoxic environment. For monocytes and macrophages, BGN and STC2 were positively correlated with monocytes, while BGN was also positively correlated with macrophages. This suggests that these genes may promote the activation of the monocyte-macrophage system, thereby enhancing immune regulation within the tumor microenvironment. Additionally, dendritic cells exhibited a strong positive correlation with BGN, suggesting an enhanced immunoregulatory role.

Among T cell subsets, CD8+ naïve T cells showed a positive correlation with BGN but negative correlations with CITED2, MT1E, MT2A, and PLAC8, implying that these genes might have influenced CD8+ T cell differentiation and maturation. For effector memory T cells, BGN was negatively correlated, whereas exhausted T cells were positively correlated with BGN and MIF, suggesting that hypoxia might have promoted an immunosuppressive state. Furthermore, regarding Treg cells, nTregs were negatively correlated with MIF but positively correlated with SLC2A1, while iTregs showed a positive correlation with DDIT4, indicating that these genes had differentially impacted T cell immunoregulation.

For NK cells, TGFBI was negatively correlated, whereas MT2A, CITED2, SRPX, PLAC8, and MT1E showed strong positive correlations, suggesting that these genes might have promoted NK cell infiltration and activation. In cytotoxic T cells, MT2A, CITED2, SRPX, and MT1E exhibited positive correlations, indicating a role in enhancing antitumor immunity. Additionally, MAIT cells were negatively correlated with BGN and STC2 but positively correlated with PLAC8. Th2 cells were negatively correlated with STC2 but positively correlated with SRPX, indicating distinct effects of different genes on specific T cell subsets.

### Developing a machine learning-based model to predict cetuximab response of CRC patients

As shown in Supplemental Figure 5 and Supplemental Table 7, the KNN model consistently outperformed other algorithms in terms of stability and generalization across the 10-fold cross-validation. This superior performance was observed on a moderate-dimensional, small-sample dataset comprising 120 samples and 16 features. The KNN model's inherent characteristics, including its low reliance on parameter tuning, balanced metric performance, and adaptability to local data distributions, contributed to its success. These advantages make KNN particularly well-suited for tasks requiring classification balance and robustness, especially in small-sample scenarios. Given its strong performance, the KNN model was selected as the final predictive model for this study, mitigating concerns of overfitting while providing a reliable and stable solution for predicting treatment response.

**Figure 6:**
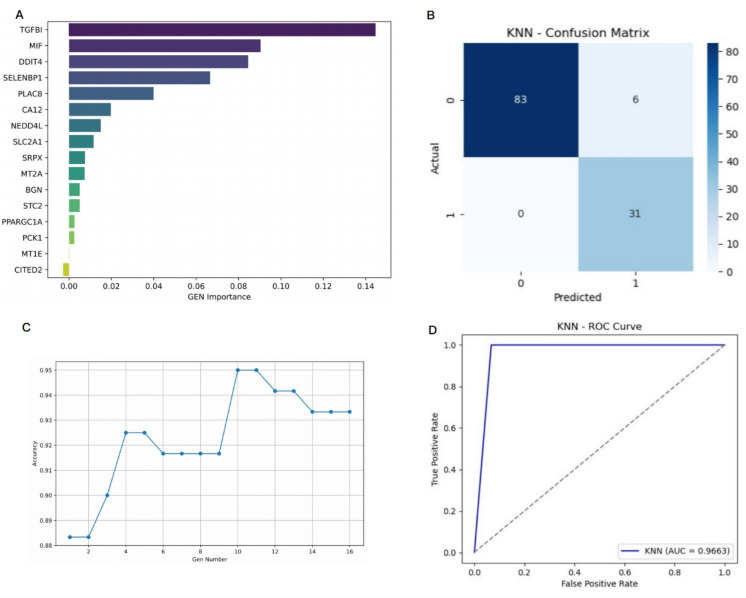
**A prediction model for the response to cetuximab in CRC patients was successfully constructed using 10 hypoxia-related DEGs.** (**A**): Horizontal axis: importance value, indicating the degree of influence of each gene on the prediction results of cetuximab treatment response. The larger the value, the more significant the contribution of the gene to the prediction results. Vertical axis: gene ID. (**B**): This figure shows the confusion matrix of the KNN algorithm, which is designed to evaluate the classification performance of the model. The horizontal axis represents the predicted category of the model, and the vertical axis represents the true category. Among them, 1 represents a sample that is sensitive to cetuximab, and 0 represents a sample that is resistant to cetuximab. (**C**): The horizontal axis represents the number of genes, and the vertical axis represents the accuracy of the prediction model. (**D**): The True Positive Rate is on the y-axis, and the False Positive Rate is on the x-axis, with the curve showing the KNN model's performance. A curve nearer to the top-left corner signifies better class distinction.

To develop a prediction model for cetuximab treatment response, we combined KNN with the Permutation Importance method for feature selection. Sixteen hypoxia-related DEGs were ranked by their importance using Permutation Importance (Figure 6A-B). From this, 10 genes significantly related to cetuximab response were selected for inclusion in the final model: CA12, DDIT4, MIF, MT2A, NEDD4L, PLAC8, SELENBP1, SLC2A1, SRPX, and TGFBI (Figure 6C). These genes demonstrated the highest prediction capacity and discriminative ability for classification tasks (Figure 6D).

The model's performance was evaluated using 10-fold cross-validation, achieving the following metrics: accuracy = 0.9500, precision = 0.8378, recall = 1.0000, F1-score = 0.9118, and area under the ROC-AUC = 0.9663. These results confirm the model's strong ability to differentiate between cetuximab-resistant and -sensitive cases. To assess the potential for overfitting, we compared the model's performance across both the training and validation sets during 10-fold cross-validation. Supplemental Table 7 indicates high consistency between training and validation metrics, with average training accuracy of 0.9650 (± 0.0064) and validation accuracy of 0.9442 (± 0.0625). Precision, recall, and F1-scores also showed minimal performance gaps, suggesting stable and robust generalization of the KNN model without significant overfitting. This consistency highlights the model's reliability on unseen data.

To further investigate the contribution of each gene, we focused on TGFBI, a top-ranked gene that plays a role in regulating extracellular matrix interactions and is implicated in cancer progression and metastasis. The impact of TGFBI on model performance was assessed by comparing the accuracy and F1 scores of the model with and without this gene. As shown in Supplemental Figure 6, the model with TGFBI outperformed the version without, achieving a validation accuracy of 0.9442 and an F1 score of 0.9422, compared to 0.8788 accuracy and 0.8916 F1 score without TGFBI. While recall remained high in both versions, precision dropped significantly (from 0.9381 to 0.8433) when TGFBI was excluded, indicating an increase in false positive predictions. This suggests that TGFBI enhances the model's ability to discriminate between cetuximab-sensitive and -resistant cases by providing biologically relevant information, thereby improving classification performance.

## Discussion

This study systematically identified and characterized hypoxia-related genes with significant roles in CRC, providing novel insights into their functional relevance, prognostic significance, and predictive potential for cetuximab treatment efficacy. Transcriptomic analyses of the GSE106582 and GSE83889 datasets identified 417 overlapping DEGs, including 16 hypoxia-related DEGs. Among these, 6 genes (BGN, DDIT4, MIF, SLC2A1, STC2, and TGFBI) were consistently upregulated, while 10 (CA12, CITED2, MT1E, MT2A, NEDD4L, PCK1, PLAC8, PPARGC1A, SELENBP1, and SRPX) were downregulated at the mRNA level. The research demonstrated that these genes were associated with roles in multiple signaling pathways related to CRC and appeared to influence the immune microenvironment. Hypoxia-related DEGs involvement had a notable impact on the OS, RFS, and PPS of CRC patients. Therefore, a combination of the KNN algorithm and Permutation Importance method were utilized to perform feature selection and construct a prediction model for cetuximab treatment response. Furthermore, 10 significant gene features (CA12, DDIT4, MIF, MT2A, NEDD4L, PLAC8, SELENBP1, SLC2A1, SRPX, and TGFBI) were selected for constructing the prediction model of cetuximab response. The model was evaluated using 10-fold cross-validation, achieving high performance metrics: accuracy (0.9500), precision (0.8378), recall (1.0000), F1-score (0.9118), and ROC-AUC score (0.9663). These results indicated excellent classification performance in distinguishing cetuximab resistant from sensitive cases.

The biological mechanisms underlying cetuximab response were further elucidated. Cetuximab is a monoclonal antibody targeting EGFR that inhibited tumor cell growth and promoted apoptosis by blocking the EGFR signaling pathway. However, resistance limited its clinical application 30. Resistance was associated with various gene mutations and aberrant activation of signaling pathways, including RAS/RAF/PIK3CA, PI3K/AKT/mTOR, Wnt/β-catenin, c-MET/HGF, and RAS-MAPK 10. Hypoxia-related genes may influence cetuximab efficacy by potentially modulating these pathways. EMT enhanced the invasiveness of cancer cells and was closely linked to cetuximab resistance 31-33. Studies show that hypoxia-related genes SRPX, BGN, CITED2, and MT2A expression is correlated with increased EMT pathway activity, suggesting a potential mechanism for promoting resistance to cetuximab. Additionally, in relation to cell cycle and DNA damage repair, higher expression of BGN was associated with decreased activity in both pathways, while higher expression of CA12, DDIT4, and MT2A was also correlated with decreased DNA damage repair activity. Given that the EGFR signaling pathway played a key role in promoting cell cycle progression and DNA repair 34, the regulatory associations of these genes might affect tumor cell sensitivity to EGFR blockade, potentially impacting cetuximab efficacy. Regarding apoptosis signaling, higher expression of MT1E and MT2A was associated with increased apoptosis pathway activity, whereas higher expression of PCK1 and TGFBI was associated with decreased activity. Since apoptosis was a crucial mechanism through which cetuximab induced tumor cell death 30, MT1E and MT2A expression might correlate with enhanced drug sensitivity, while PCK1 and TGFBI expression might be linked to resistance. Therefore, the expression patterns of these genes could serve as potential biomarkers for cetuximab sensitivity or resistance, providing insights for personalized treatment strategies. Future studies could explore combination therapy approaches targeting these genes to improve clinical outcomes of cetuximab treatment.

Immunological analysis indicated that hypoxia-related genes might have regulated cetuximab efficacy by potentially influencing immune cell infiltration and the tumor microenvironment 35. Expression of BGN, DDIT4, MIF, TGFBI, and STC2 was negatively correlated with B cell infiltration, suggesting a potential suppressive effect on B cell function that could impact antibody-mediated immunity. Meanwhile, expression of these genes was positively correlated with macrophage infiltration, possibly contributing to altered tumor-associated macrophage phenotypes, which might impact cetuximab response 36. T cell exhaustion was a key mechanism of tumor immune evasion 37. Expression of BGN and MIF was positively correlated with exhausted T cells, suggesting a potential role in T cell dysfunction and resistance. Treg cells promoted an immunosuppressive microenvironment by inhibiting CD8+ cytotoxic T cells 38. Expression of SLC2A1 and DDIT4 was positively correlated with Treg infiltration, potentially enhancing immune suppression. NK cells and CD8+ cytotoxic T cells played critical roles in tumor immune surveillance and antibody-dependent cellular cytotoxicity 39-41. Expression of MT2A, CITED2, SRPX, PLAC8, and MT1E was positively correlated with NK and cytotoxic T cell infiltration, potentially enhancing antitumor immunity and improving the potential for cetuximab efficacy. These findings highlighted the complex associations of these genes with oxidative stress and the immune microenvironment, underscoring their significance in CRC progression and therapy resistance.

Our hypoxia-related gene signature offers certain advantages over established cetuximab response predictors. While consensus molecular subtypes (CMS) provide valuable insights into CRC biology 42, they may lack the specificity required for accurately predicting responses to anti-EGFR therapies. Similarly, recent biomarker studies focused on phosphoproteomics 43, miRNA regulators (e.g., miR-196b) 44, or receptor internalization have contributed important findings 45, 46, but these approaches can face challenges in clinical translation due to their technical complexity or limitations related to single-gene markers. In contrast, our 10-gene hypoxia signature integrates multiple resistance mechanisms, including EMT, cell cycle dysregulation, and immune escape, which have previously been implicated in cetuximab resistance 46. In addition, hypoxia has been shown to modulate immune cell infiltration and activity, further strengthening its role in therapeutic resistance 47. By targeting these hypoxia-related genes, our model provides a more accurate and clinically useful tool for predicting the efficacy of cetuximab. In clinical practice, bevacizumab, another commonly used targeted therapy, exerts anti-tumor effects by inhibiting VEGF-mediated angiogenesis. However, studies have shown that this anti-angiogenic effect may exacerbate tumor hypoxia 48, 49, thereby influencing subsequent cetuximab treatment outcomes. Based on our model, it could serve as a tool for stratifying patients' cetuximab treatment response after bevacizumab therapy, helping to identify those whose treatment may be compromised due to tumor hypoxia, thus optimizing therapeutic strategies.

In future studies, we will validate these predictive models in larger, independent cohorts and clinical settings. Incorporating additional features, such as genomic mutations, immune landscape data, or clinical parameters, could further improve the predictive accuracy and clinical utility of these models. Moreover, applying these models to guide combination therapies, such as pairing cetuximab with hypoxia-targeted agents or immunomodulators, will make optimizing treatment regimens and overcoming drug resistance a reality.

## Conclusion

This study highlights the pivotal roles of hypoxia-related DEGs in cetuximab response, providing a foundation for the development of predictive models and personalized therapeutic strategies in CRC. By combining machine learning tools with biological insights, this research advances the understanding of hypoxia-driven mechanisms in CRC and offers new directions for precision medicine approaches aimed at improving patient outcomes. Further exploration of these strategies in clinical settings is essential to translate these findings into meaningful therapeutic advances.

## Supplementary Material

Supplementary figures and tables 5-7.

Supplementary table 1.

Supplementary table 2.

Supplementary table 3.

Supplementary table 4.
