# Supplementary material for: A Machine Learning-Based Hypoxia-Related Gene Signatures to Facilitate Prediction of Cetuximab Response in Patients with Colorectal Cancer
Source: Int J Med Sci. 2025 Aug 11;22(14):3749–62. doi: 10.7150/ijms.114833 (PMC12434828; doi:10.7150/ijms.114833)
Supplement: Supplementary file 1 — Supplementary figures and tables 5-7. [file ijmsv22p3749s1.pdf]

Supplemental Figure lists

Supplemental Figure 1. Functional Enrichment Analysis of Upregulated and Downregulated Genes in CRC from the GSE106582 Dataset

Panel A shows the most enriched GO terms for upregulated genes in the GSE106582 dataset, categorized by biological process (red), molecular function (blue), and cellular component (orange). Panel B displays the KEGG pathway enrichment for upregulated genes, with the size and color of each dot indicating gene count and significance (-log10(*p*-value)). Panel C presents the most enriched GO terms for downregulated genes in the GSE106582 dataset, categorized similarly to Panel A. Panel D shows the KEGG pathway enrichment for downregulated genes, with dot size and color representing gene count and significance, respectively.

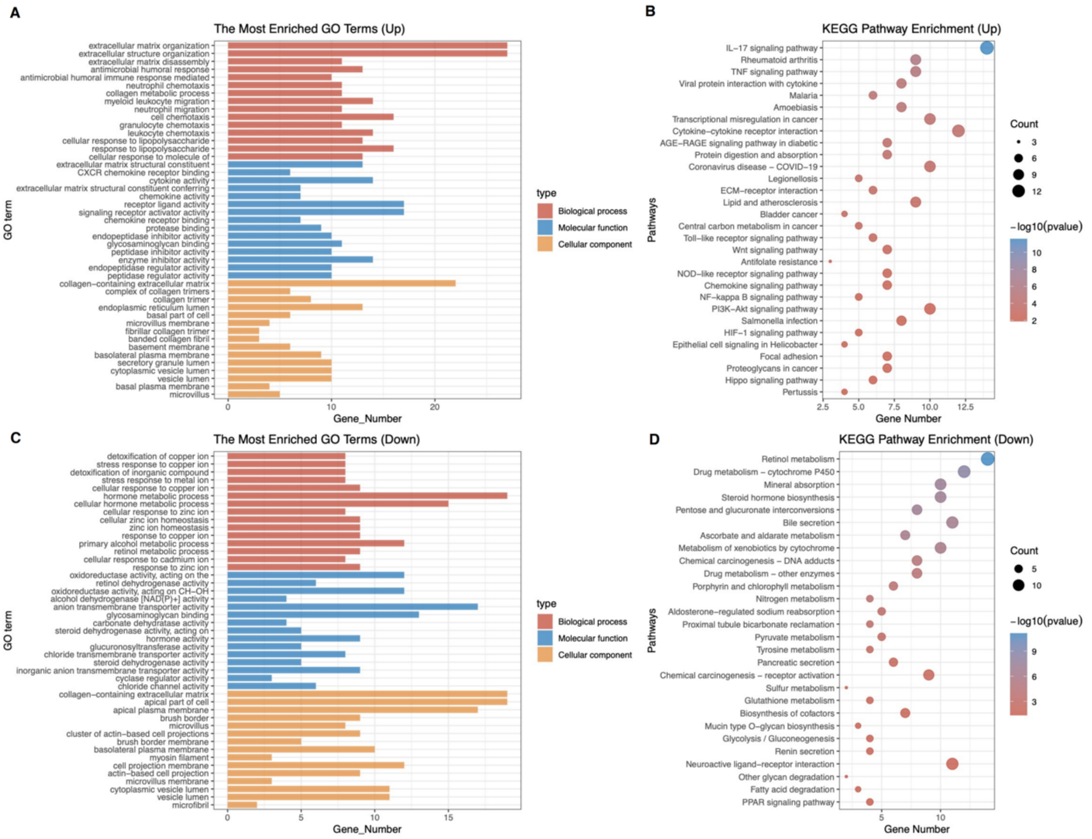

Supplemental Figure 2. Functional Enrichment Analysis of Upregulated and Downregulated Genes in CRC from the GSE83889 Dataset

Panel A shows the most enriched GO terms for upregulated genes in the GSE83889 dataset, categorized by biological process (red), molecular function (blue), and cellular component (orange). Panel B displays the KEGG pathway enrichment for upregulated genes, with the size and color of each dot indicating gene count and significance (-log10(*p*-value)). Panel C presents the most enriched GO terms for downregulated genes in the GSE83889 dataset, categorized similarly to Panel A.

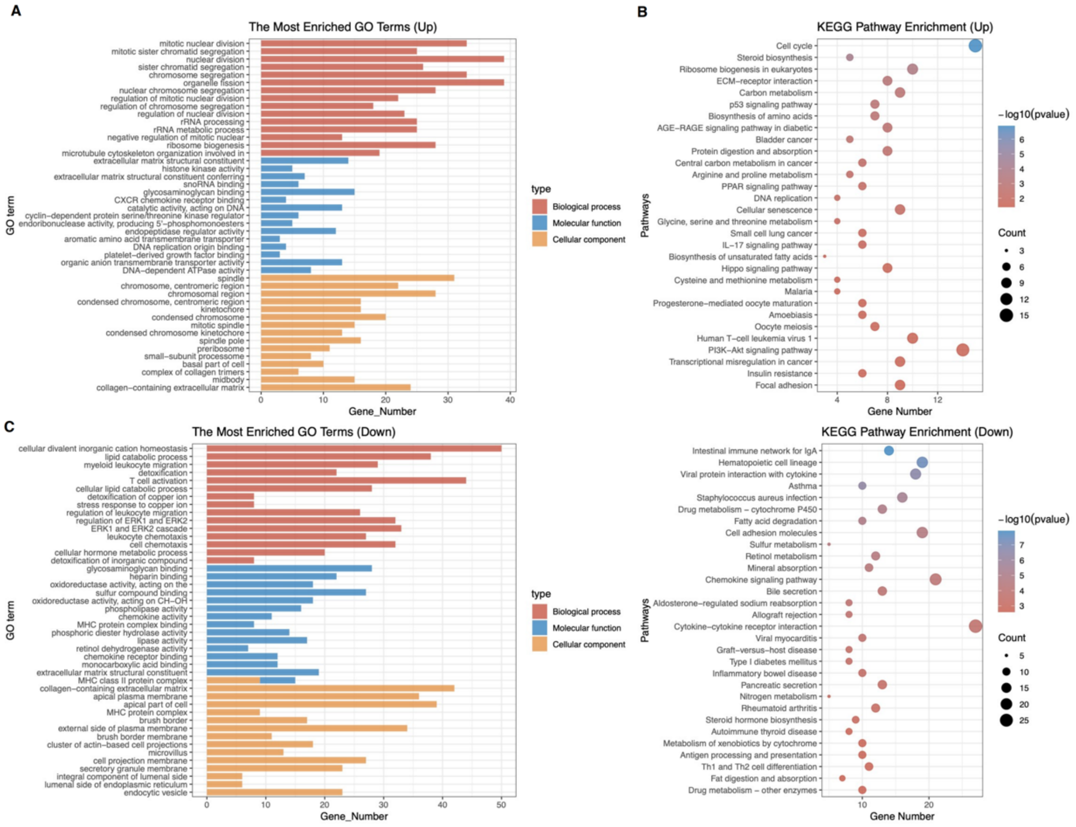

Supplemental Figure 3. Prognostic Analysis of Hypoxia-Related DEGs in CRC

This figure presents Kaplan-Meier survival curves for 9 hypoxia-related DEGs significantly associated with OS, RFS, and PPS in CRC. The survival curves compare patients with high vs. low expression levels, with the red line representing high expression and the black line representing low expression. HR and log-rank p-values are shown for each gene. The X-axis represents time in months, and the Y-axis represents survival probability. The figure includes the following genes: *CITED2*, *DDIT4*, *MT2A*, *PLAC8*, *STC2*, *MTE1*, *PPARGC1A*, *CA12*, and *PCK1*.

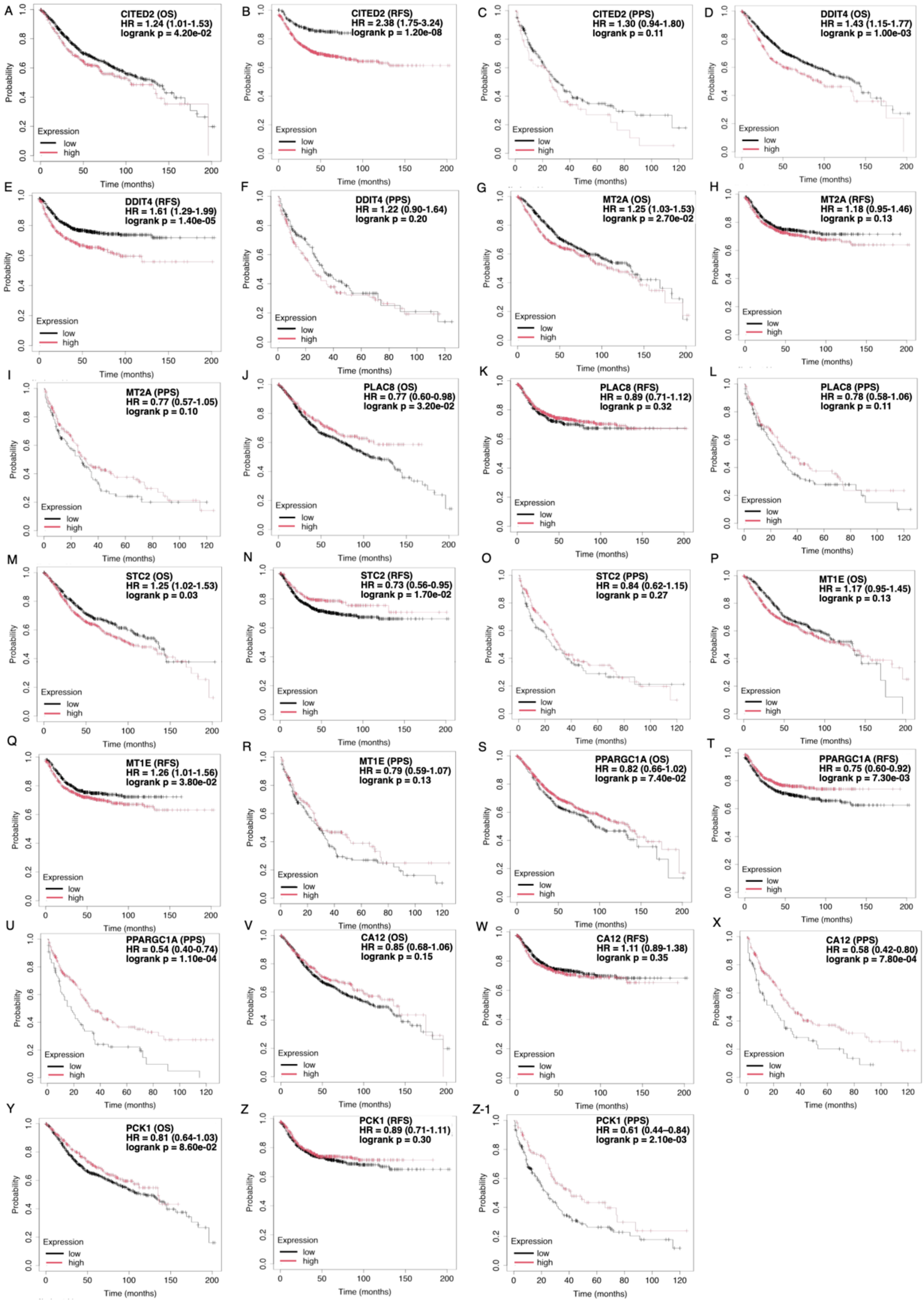

Supplemental Figure 4. Correlation between Hypoxia-Related DEGs and Immune Cell Infiltration in CRC

This figure displays the correlation between hypoxia-related DEGs (listed on the left) and various immune cell types (listed at the bottom) in CRC. Each circle represents a correlation, with the color intensity indicating the strength of the correlation: red for positive correlations and purple for negative correlations. The size of the circles corresponds to the significance of the correlation, with larger circles representing a FDR <= 0.05, and smaller circles representing FDR > 0.05. The correlation coefficients range from -0.5 (negative correlation) to 0.7 (positive correlation).

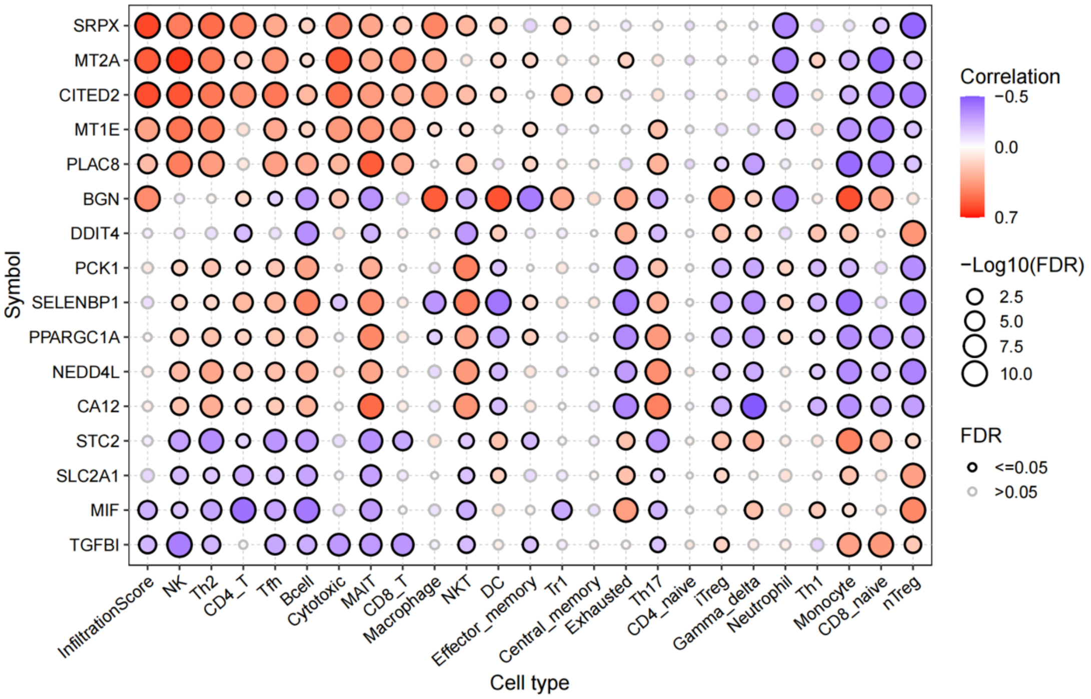

Supplemental Figure 5. Performance Metrics of Different Machine Learning Models Across 10-Fold Cross-Validation.

(A) KNN; (B) Random Forests; (C) SVM; (D) XGBoost. Each panel shows the evaluation metrics (accuracy, precision, recall, and F1-score) for the respective machine learning model across the 10 different folds of cross-validation. The lines represent the different performance metrics, allowing comparison of how each algorithm performs across various data splits.

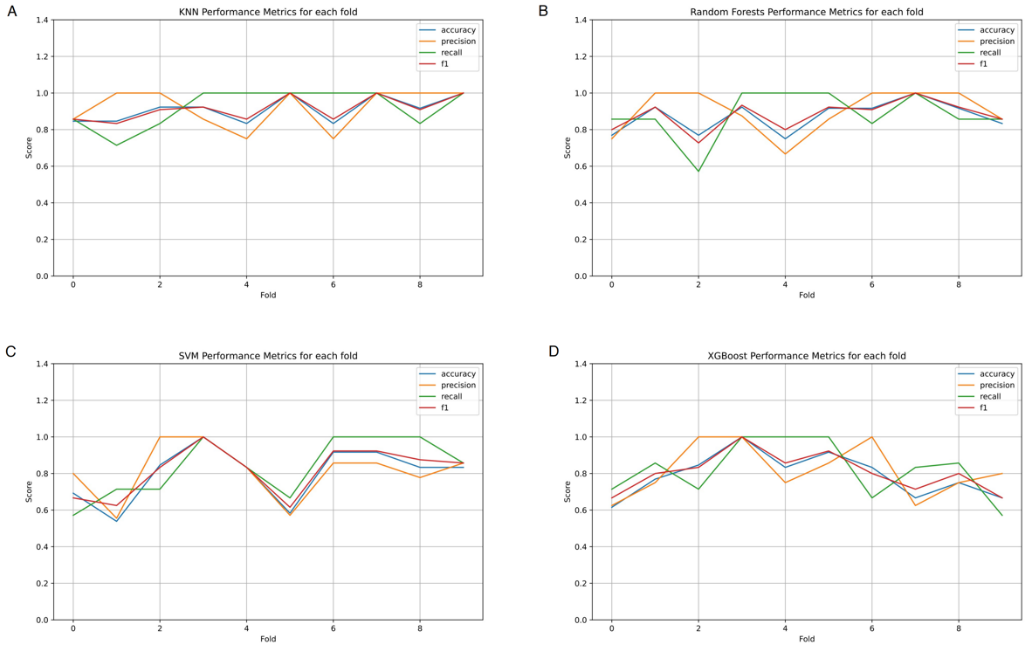

Supplemental Figure 6. Performance Comparison of the Model with and without *TGFBI*

This figure illustrates the performance metrics (Accuracy, Precision, Recall, and F1 Score) of the predictive model during training and validation, both with and without the inclusion of the *TGFBI* gene. The bars represent the performance scores for the following categories: Train (with *TGFBI*): Model performance during training with *TGFBI* included. Train (without *TGFBI*): Model performance during training without *TGFBI*. Validation (with *TGFBI*): Model performance during validation with *TGFBI* included. Validation (without *TGFBI)*: Model performance during validation without *TGFBI*. Each performance metric is shown as a separate set of bars, allowing for a direct comparison between the models with and without *TGFBI* across different evaluation criteria.

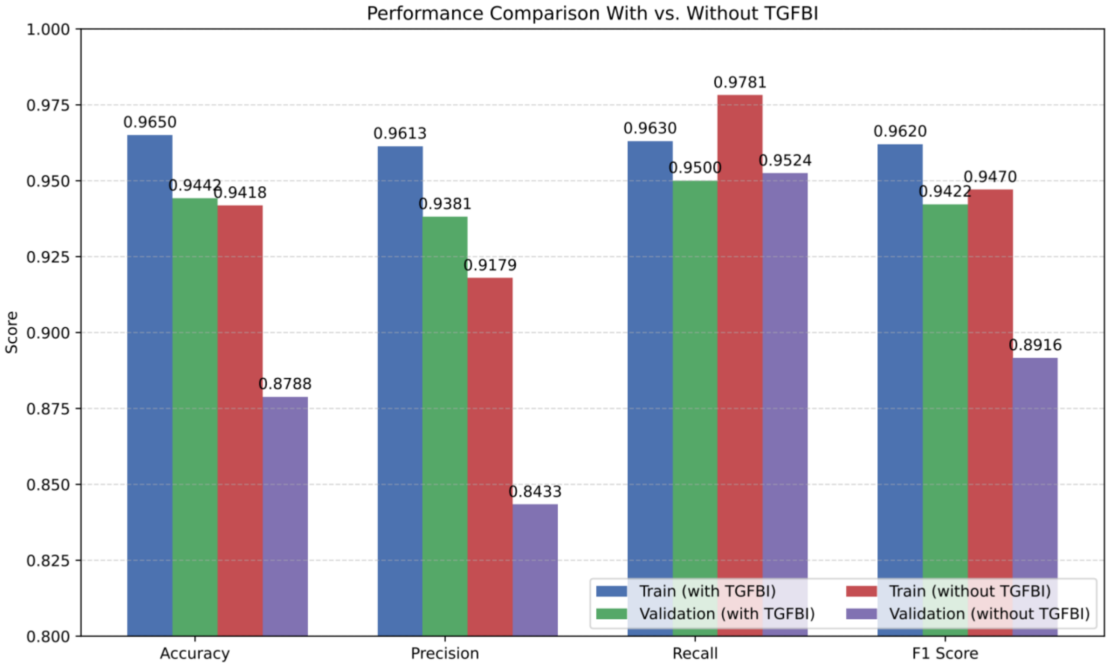

Supplemental Table lists

Supplemental Table 5. Prognostic Significance of 16 Hypoxia-related DEGs in CRC

Gene (Affimetrix ID)	OS		RFS		PPS	
	HR (95% CI)	Logrank *p*	HR (95% CI)	Logrank *p*	HR (95% CI)	Logrank *p*
BGN (201261_x_at) [a, b, c]	1.64 (1.33 − 2.01)	2.60e-06	1.88 (1.52 − 2.32)	3.80e-09	1.51 (1.10 − 2.08)	9.70e-03
CA12 (204508_s_at) [a]	0.85 (0.68 − 1.06)	1.50e-01	1.11 (0.89 − 1.38)	3.50e-01	0.58 (0.42 − 0.8)	7.80e-04
CITED2 (207980_s_at) [a, b]	1.24 (1.01 − 1.53)	4.00e-02	2.38 (1.75 − 3.24)	1.20e-08	1.30 (0.94 − 1.80)	1.10e-01
DDIT4 (202887_s_at) [a, b]	1.43 (1.15 − 1.77)	1.00e-03	1.61 (1.29 − 1.99)	1.40e-05	1.22 (0.90 − 1.64)	2.00e-01
MIF (221262_s_at) [a, b, c]	1.30 (1.06 − 1.61)	1.40e-02	1.31 (1.05 − 1.63)	1.60e-02	0.72 (0.53 − 0.97)	2.90e-02
MT1E (212859_x_at) [b]	1.17 (0.95 − 1.45)	1.30e-01	1.26 (1.01 − 1.56)	3.80e-02	0.79 (0.59 − 1.07)	1.30e-01
MT2A (212185_x_at) [a]	1.25 (1.03 − 1.53)	2.70e-02	1.18 (0.95 − 1.46)	1.30e-01	0.77 (0.57 − 1.05)	1.00e-01
NEDD4L (212445_s_at) [a, b, c]	0.75 (0.60 − 0.93)	9.40e-03	0.59 (0.47 − 0.73)	1.30e-06	0.53 (0.39 − 0.71)	2.40e-05
PCK1 (208383_s_at) [c]	0.81 (0.64 − 1.03)	8.60e-02	0.89 (0.71 − 1.11)	3.00e-01	0.61 (0.44 − 0.84)	2.10e-03
PLAC8 (219014_at) [a]	0.77 (0.60 − 0.98)	3.20e-02	0.89 (0.71 − 1.12)	3.20e-01	0.78 (0.58 − 1.06)	1.10e-01
PPARGC1A (219195_at) [b, c]	0.82 (0.66 − 1.02)	7.40e-02	0.75 (0.60 − 0.92)	7.30e-03	0.54 (0.40 − 0.74)	1.10e-04
SELENBP1 (214433_s_at) [a, b, c]	0.65 (0.53 − 0.81)	1.40e-04	0.56 (0.43 − 0.73)	1.60e-05	0.64 (0.47 − 0.86)	2.90e-03
SLC2A1 (201249_at) [a, b, c]	1.49 (1.19 − 1.88)	5.90e-04	1.53 (1.24 − 1.90)	8.80e-05	1.46 (1.07 − 2.00)	1.70e-02
SRPX (204955_at) [a, b, c]	1.55 (1.27 − 1.89)	1.70e–05	1.49 (1.20 − 1.84)	2.10e-04	1.64 (1.21 − 2.21)	1.10e-03
STC2 (203438_at) [a, b]	1.25 (1.02 − 1.53)	3.00e-02	0.73 (0.56 − 0.95)	1.70e-02	0.84 (0.62 − 1.15)	2.70e-01
TGFBI (201506_at) [a, b, c]	1.39 (1.14 − 1.70)	1.00e-03	1.45 (1.17 − 1.79)	6.00e–04	1.45 (1.08 − 1.96)	1.40e-02

a: Indicates significant association with OS ($p < 0.05$); b: Indicates significant association with RFS ($p < 0.05$); c: Indicates significant association with PPS ($p < 0.05$).

Supplemental Table 6. Hypoxia-Related DEGs Correlate with Immunosuppressive and Cytotoxic Immune Cell Infiltration in CRC

Number	Symbol	Cell_Type	correlation	*p*-value	adjusted *p*-value
1	*BGN*	Bcell	-0.31	1.62E-08	1.63E-07
2	*BGN*	CD8_naive	0.34	2.37E-10	4.28E-09
3	*BGN*	DC	0.58	4.30E-31	1.06E-28
4	*BGN*	Effector_memory	-0.40	9.24E-14	2.19E-11
5	*BGN*	Exhausted	0.32	3.16E-09	5.16E-08
6	*BGN*	InfiltrationScore	0.41	4.86E-15	3.80E-14
7	*BGN*	MAIT	-0.33	1.01E-09	7.42E-09
8	*BGN*	Macrophage	0.56	3.50E-28	1.78E-26
9	*BGN*	Monocyte	0.59	1.13E-31	2.49E-28
10	*BGN*	Neutrophil	-0.39	3.31E-13	3.67E-12
11	*BGN*	Tr1	0.32	4.62E-09	3.97E-08
12	*BGN*	iTreg	0.43	1.58E-16	9.54E-15
13	*CA12*	Exhausted	-0.37	7.86E-12	2.71E-10
14	*CA12*	Gamma_delta	-0.50	4.12E-22	2.05E-18
15	*CA12*	MAIT	0.52	9.73E-24	1.78E-21
16	*CA12*	Monocyte	-0.33	6.32E-10	8.19E-09
17	*CA12*	NKT	0.38	1.06E-12	2.82E-11
18	*CA12*	Th17	0.45	1.41E-17	6.26E-15
19	*CITED2*	CD4_T	0.39	1.57E-13	1.04E-12
20	*CITED2*	CD8_naive	-0.39	3.17E-13	1.39E-11
21	*CITED2*	Cytotoxic	0.50	9.69E-22	2.83E-20
22	*CITED2*	InfiltrationScore	0.59	1.55E-32	3.08E-31
23	*CITED2*	MAIT	0.36	1.48E-11	1.66E-10
24	*CITED2*	Macrophage	0.37	2.27E-12	2.75E-11
25	*CITED2*	NK	0.58	2.29E-30	1.26E-28
26	*CITED2*	Neutrophil	-0.39	3.15E-13	3.50E-12
27	*CITED2*	Tfh	0.48	3.44E-20	1.22E-18
28	*CITED2*	Th2	0.47	1.24E-19	2.28E-18
29	*CITED2*	nTreg	-0.39	2.93E-13	2.78E-12
30	*DDIT4*	Bcell	-0.34	3.79E-10	6.68E-09
31	*DDIT4*	NKT	-0.31	9.69E-09	9.21E-08
32	*DDIT4*	nTreg	0.38	9.61E-13	8.37E-12
33	*MIF*	Bcell	-0.41	1.87E-14	1.28E-12
34	*MIF*	CD4_T	-0.43	6.14E-16	5.29E-15
35	*MIF*	Exhausted	0.35	6.45E-11	1.79E-09

36	*MIF*	nTreg	0.43	7.49E-16	1.13E-14
37	*MT1E*	CD8_T	0.35	6.44E-11	1.58E-09
38	*MT1E*	CD8_naive	-0.39	2.15E-13	9.89E-12
39	*MT1E*	Cytotoxic	0.37	5.90E-12	6.77E-11
40	*MT1E*	InfiltrationScore	0.33	6.39E-10	3.21E-09
41	*MT1E*	MAIT	0.38	6.24E-13	9.72E-12
42	*MT1E*	Monocyte	-0.33	1.36E-09	1.60E-08
43	*MT1E*	NK	0.49	3.53E-21	8.45E-20
44	*MT1E*	Tfh	0.31	1.25E-08	7.93E-08
45	*MT1E*	Th2	0.44	2.74E-17	3.45E-16
46	*MT2A*	CD8_T	0.42	3.61E-15	1.54E-13
47	*MT2A*	CD8_naive	-0.44	7.37E-17	1.11E-14
48	*MT2A*	Cytotoxic	0.56	9.28E-29	3.91E-27
49	*MT2A*	InfiltrationScore	0.56	5.83E-28	9.14E-27
50	*MT2A*	MAIT	0.31	9.90E-09	5.90E-08
51	*MT2A*	Macrophage	0.32	1.74E-09	1.58E-08
52	*MT2A*	NK	0.64	1.35E-38	1.45E-36
53	*MT2A*	Neutrophil	-0.38	9.37E-13	9.87E-12
54	*MT2A*	Tfh	0.38	6.74E-13	9.98E-12
55	*MT2A*	Th2	0.47	5.12E-19	8.48E-18
56	*NEDD4L*	MAIT	0.32	2.84E-09	1.89E-08
57	*NEDD4L*	Monocyte	-0.35	1.19E-10	1.93E-09
58	*NEDD4L*	NKT	0.36	9.62E-12	1.99E-10
59	*NEDD4L*	Th17	0.40	3.16E-14	4.36E-12
60	*NEDD4L*	Th2	0.32	3.34E-09	1.43E-08
61	*NEDD4L*	nTreg	-0.37	2.77E-12	2.24E-11
62	*PCK1*	Bcell	0.33	8.00E-10	1.25E-08
63	*PCK1*	Exhausted	-0.34	2.58E-10	5.88E-09
64	*PCK1*	NKT	0.45	1.17E-17	1.20E-15
65	*PCK1*	nTreg	-0.34	1.94E-10	1.17E-09
66	*PLAC8*	Bcell	0.31	1.65E-08	1.66E-07
67	*PLAC8*	CD8_naive	-0.40	5.10E-14	2.90E-12
68	*PLAC8*	MAIT	0.55	6.65E-28	3.15E-25
69	*PLAC8*	Monocyte	-0.45	1.97E-17	2.35E-15
70	*PLAC8*	NK	0.46	1.94E-18	3.28E-17
71	*PLAC8*	Tfh	0.35	1.10E-10	1.07E-09
72	*PLAC8*	Th2	0.36	1.54E-11	8.86E-11
73	*PPARGC1A*	CD8_naive	-0.33	8.18E-10	1.29E-08
74	*PPARGC1A*	Exhausted	-0.35	5.17E-11	1.47E-09

75	*PPARGC1A*	MAIT	0.42	1.54E-15	4.38E-14
76	*PPARGC1A*	Monocyte	-0.34	2.55E-10	3.73E-09
77	*PPARGC1A*	NKT	0.32	4.82E-09	4.96E-08
78	*PPARGC1A*	Th17	0.37	7.88E-12	4.25E-10
79	*SELENBP1*	Bcell	0.43	3.30E-16	3.71E-14
80	*SELENBP1*	DC	-0.42	3.33E-15	1.38E-13
81	*SELENBP1*	Exhausted	-0.40	4.62E-14	2.94E-12
82	*SELENBP1*	Gamma_delta	-0.33	8.88E-10	3.00E-08
83	*SELENBP1*	MAIT	0.40	3.32E-14	7.15E-13
84	*SELENBP1*	Macrophage	-0.32	5.26E-09	4.55E-08
85	*SELENBP1*	Monocyte	-0.43	3.25E-16	2.72E-14
86	*SELENBP1*	NKT	0.46	1.63E-18	2.32E-16
87	*SELENBP1*	nTreg	-0.39	4.41E-13	4.06E-12
88	*SLC2A1*	nTreg	0.34	2.34E-10	1.39E-09
89	*SRPX*	CD4_T	0.43	1.47E-16	1.35E-15
90	*SRPX*	Cytotoxic	0.42	3.19E-15	5.54E-14
91	*SRPX*	InfiltrationScore	0.61	1.27E-34	2.80E-33
92	*SRPX*	MAIT	0.32	4.20E-09	2.70E-08
93	*SRPX*	Macrophage	0.43	4.91E-16	8.30E-15
94	*SRPX*	NK	0.46	9.55E-19	1.67E-17
95	*SRPX*	Neutrophil	-0.37	6.69E-12	6.46E-11
96	*SRPX*	Tfh	0.31	1.04E-08	6.66E-08
97	*SRPX*	Th2	0.51	6.03E-23	1.93E-21
98	*SRPX*	nTreg	-0.46	1.97E-18	4.88E-17
99	*STC2*	Bcell	-0.31	1.27E-08	1.33E-07
100	*STC2*	MAIT	-0.33	6.03E-10	4.65E-09
101	*STC2*	Monocyte	0.45	2.21E-17	2.57E-15
102	*STC2*	Tfh	-0.32	3.67E-09	2.58E-08
103	*STC2*	Th17	-0.32	2.26E-09	4.95E-08
104	*STC2*	Th2	-0.34	1.55E-10	7.83E-10
105	*TGFBI*	CD8_T	-0.32	4.63E-09	8.20E-08
106	*TGFBI*	CD8_naive	0.36	1.31E-11	3.54E-10
107	*TGFBI*	Cytotoxic	-0.31	1.40E-08	9.31E-08
108	*TGFBI*	MAIT	-0.30	2.00E-08	1.12E-07
109	*TGFBI*	Monocyte	0.34	4.00E-10	5.51E-09
110	*TGFBI*	NK	-0.39	3.72E-13	2.92E-12

Supplemental Table 7. Average Performance Metrics of KNN Across 10-Fold Cross-Validation

Metric	Training set	Validation set
Accuracy	0.9650 ± 0.0064	0.9442 ± 0.0625
Precision	0.9613 ± 0.0119	0.9381 ± 0.0762
Recall	0.9630 ± 0.0105	0.9500 ± 0.0764
F1 Score	0.9620 ± 0.0067	0.9422 ± 0.0650
